# ‘Chronomics’ in ICU: circadian aspects of immune response and therapeutic perspectives in the critically ill

**DOI:** 10.1186/2197-425X-2-18

**Published:** 2014-05-14

**Authors:** Vasilios Papaioannou, Alexandre Mebazaa, Benoît Plaud, Matthieu Legrand

**Affiliations:** Department of Anesthesiology and Critical Care and Burn Unit, Hôpitaux Universitaire St-Louis-Lariboisière, Assistance Publique-Hôpitaux de Paris, University of Paris Diderot, U942 INSERM, Paris, 75475 France; Intensive Care Unit, Alexandroupolis University Hospital, Democritus University of Thrace, School of Medicine, Dragana, Alexandroupolis, 68100 Greece

**Keywords:** Circadian rhythm, Cortisol, Critical illness, Cytokines, Melatonin, Pineal gland, Sepsis

## Abstract

Complex interrelations exist between the master central clock, located in the suprachiasmatic nuclei of the hypothalamus, and several peripheral clocks, such as those found in different immune cells of the body. Moreover, external factors that are called ‘timekeepers’, such as light/dark and sleep/wake cycles, interact with internal clocks by synchronizing their different oscillation phases. Chronobiology is the science that studies biologic rhythms exhibiting recurrent cyclic behavior. Circadian rhythms have a duration of approximately 24 h and can be assessed through chronobiologic analysis of time series of melatonin, cortisol, and temperature. Critically ill patients experience severe circadian deregulation due to not only the lack of effective timekeepers in the intensive care unit (ICU) environment but also systemic inflammation. The latter has been found in both animal and human studies to disrupt circadian rhythmicity of all measured biomarkers. The aims of this article are to describe circadian physiology during acute stress and to discuss the effects of ICU milieu upon circadian rhythms, in order to emphasize the value of considering circadian-immune disturbance as a potential tool for personalized treatment. Thus, besides neoplastic processes, critical illness could be linked to what has been referred as ‘chronomics’: timing and rhythm. In addition, different therapeutic perspectives will be presented in association with environmental approaches that could restore circadian connection and hasten physical recovery.

## Review

### Introduction

Circadian rhythms refer to self-sustained fluctuations with a period of approximately (*circa*) 1 day (*diem*) in various physiological processes. Circadian rhythmicity is observed for many hormones in circulation (i.e., corticosteroids) as well as for circulating immune cells and cytokines [1, 2]. Ten circadian clock genes have been identified in human peripheral tissues so far, including Period (Per-1-3), Crypto-chrome (Cry-1 and Cry-2), Clock, and Bmal1, which coordinate with the master clock located in the suprachiasmatic nuclei (SCN) of the anterior hypothalamus [3].

**Figure 1:**
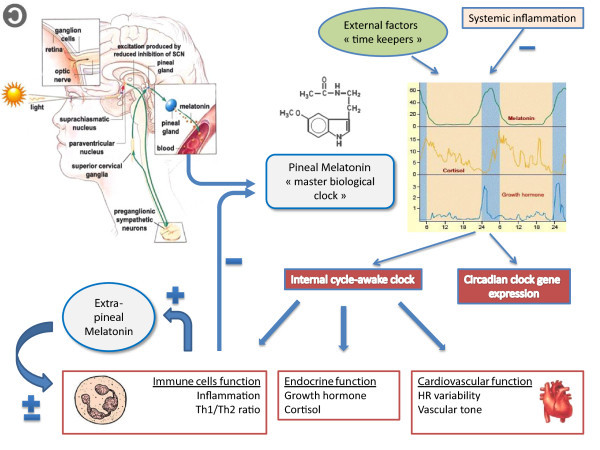
**Melatonin: the ‘master biological clock’.** Non-visual effects of light are mediated through specific retinal ganglion cells which subsequently activate SCN neurons. As a result, SCN inhibits the pineal production of melatonin during daytime through a polysynaptic pathway including paraventricular nucleus (PVN), superior cervical ganglia, and preganglionic sympathetic neurons of the lateral horn of the spinal cord. The pineal melatonin is considered the master biological clock that synchronizes the circadian rhythms of different clock genes throughout the body with different external ‘timekeepers’, such as light/dark cycles. Furthermore, the SCN-PVN network is responsible for 24-h period fluctuations of both sympathetic and parasympathetic tone, estimated with heart rate variability analysis, and for circadian oscillations of immunity and endocrine function. During inflammation, circadian rhythms of different hormones are disrupted, whereas immune cells in the periphery suppress melatonin's nocturnal surge through TNF-α and produce melatonin themselves. This extrapineal melatonin acts on a paracrine manner and exhibits both pro- and anti-inflammatory properties, depending on time phase and severity of stress. SCN, suprachiasmatic nucleus; PVN, paraventricular nucleus. Figures are reproduced from the free website: ‘The brain from top to bottom’, according to its copyleft policy (http://thebrain.mcgill.ca/flash/pop/popcopy/popcopy.html).

In mammals, the circadian system is composed of many individual, tissue-specific clocks with their phase being controlled by the master circadian pacemaker of SCN [1]. SCN neurons control clock genes throughout the body by controlling two major communication channels, the endocrine system and the autonomic nervous system (ANS). The recent discovery of a novel third type of retinal photoreceptor, other than rods and cones, provided evidence of a pathway mediating non-visual effects of light [4]. Subsequent signals are directed towards SCN neurons through the retinohypothalamic tract and synchronize them to the day/light cycle. Furthermore, connections of SCN with other hypothalamic structures allow the master clock to synchronize other clock genes in the body [5, 6]. Additionally, through sympathetic nerve projections, SCN output signals induce the release of a major internal synchronizer, the pineal substance melatonin (Figure 1) [5, 7].

Melatonin is synthesized by the pineal gland upon β adrenoreceptor stimulation of pinealocytes, increased during sleepiness, and decreased during wakefulness, and it conveys the information of nighttime to the organism. In healthy humans, melatonin secretion starts between 9:00 p.m. and 11:00 p.m., reaching peak serum levels between 1:00 a.m. and 3:00 a.m. (>40 pg/mL) and then falling to low baseline values between 7:00 a.m. and 9:00 a.m (<7 pg/mL) [8, 9]. It also plays the role of an endogenous synchronizer, which is able to stabilize circadian rhythms and maintain their mutual phase relationships. Furthermore, its rhythm is involved in the regulation of the sleep/wake cycle, sleep structure, and more generally in the temporal organization of immunity [1, 5]. The last is considered as an effective component of ‘predictive homeostasis’ since nearly all organisms have developed mechanisms for anticipating environmental changes to optimize their survival [1, 2]. In this respect, temporal organization of immune response maximizes it at the time of the day that is needed most, since exposure to microbial pathogens depends on intrinsic 24-h rhythms of the host (activity, feeding). Moreover, immune modulation by the ANS, which also displays a diurnal rhythmicity, further supports the notion of immune regulation by light/dark cycle [1, 6]. Melatonin is also considered an active anti-inflammatory molecule due to the inhibition of tumor necrosis factor α (TNF-α) production [9, 10]. In addition, melatonin has an extrapineal source since different gastrointestinal cells synthesize melatonin, which has a peripheral activity (e.g., protection against reperfusion injury in gut mucosa), through its antioxidant properties [11].

Circadian rhythms are also synchronized and maintained by different phase relationships to external factors. These rhythms persist with an identical period (light/dark, sleep/wake) or are different throughout a day. These external factors are also called ‘timekeepers’ and are considered as effective modulators for the circadian oscillator (e.g., light, feeding, ambient temperature, and stress) [12].

Several studies have demonstrated that there is a circadian rhythmicity of different components of the immune system [1, 13–15]. Moreover, it has been suggested that circadian regulation of immunity is necessary for temporal coincidence of all its different molecular steps [13–15]. Thus, circadian oscillations of lymphocyte proliferation, antigen presentation, and cytokine gene expression appear coordinated via SCN output signals. Additionally, the number of most immune cells reaches maximal values during the night and is lowered after arousal [1].

### Circadian physiology and inflammation

#### Experimental data

Different pro-inflammatory cytokines, such as TNF-α and interleukin-6 (IL-6), may cross the blood-brain barrier at leaky points (the circumventricular organs (CVO)) and induce a ‘sickness behavior’ , associated with decreased amplitude of circadian rhythmicity, such as loss of sleep/wake cycle [1, 16].

Many studies have found that the susceptibility of mice to lipopolysaccharide (LPS) and TNF-α-induced lethality varied significantly throughout the day, depending on the time of administration [17–20]. Moreover, immune response upon LPS challenge, such as cytokine production [21] or toll-like receptor 9 (TLR9) expression [22], has been shown to display circadian rhythmicity, depending on time of LPS administration. Chronic inflammation can also affect SCN output by reducing amplitude and average spiking frequency of SCN neurons [23, 24]. In addition, LPS exposure has been found to suppress mRNA expression levels of different clock genes, in both animal [25] and human studies [26, 27]. However, melatonin and cortisol circadian rhythms were not affected by LPS (Table 1). It has been suggested that centrally regulated hormones' circadian rhythmicity and peripheral clock gene expression are independently regulated during sepsis, reflecting an uncoupling between central and peripheral oscillators during systemic inflammation [28].

**Table 1:** **Immune-circadian connection: experimental studies**

Author	Study design	Major outcome
Haldberg et al. [17]	Susceptibility of mice to *Escherichia coli* endotoxin-induced lethality	Lethality varied significantly throughout the day, depending on the time when mice were challenged
Hrushesky et al. [18]	Effect of time of TNF-α administration on lethal toxicity in mice	Nine-fold variation of lethality being greatest during night and particularly before awakening
Keller et al. [21]	Splenocytes from mice, isolated at various times of the day, were challenged with LPS	Circadian rhythmicity of TNF-α and IL-6 secretion was found. More than 8% of the peritoneal macrophage transcriptome oscillates in a circadian function autonomically and depends on time of LPS challenge
Silver et al. [22]	Toll-like receptor 9 (TLR9) expressed in peritoneal macrophages were estimated for circadian rhythmicity in a mouse model of sepsis	Vaccination with TLR9 ligand as adjuvant at the time of enhanced TLR9 responsiveness induced an improved adaptive immune response many weeks later. Moreover, disease severity was dependent on the timing of sepsis induction, coinciding with daily changes in TLR9 expression
Kwak et al. [24]	Study of the long-term effects of INF-γ on SCN neurons by treating dispersed rat SCN neurons with INF-γ for a 4-week period	Firing of SCN neurons and rhythmic expression of clock gene *Per1* exhibited a lower average spiking frequency with reduced amplitude and an irregular firing pattern, in relation with controls
Okada et al. [25]	LPS effects on mRNA expression of clock genes in rats	mRNA expression levels of different clock genes, such as *Per 1* and *Per 2*, both in the liver and SCN neurons on day 1, were suppressed with an expression nadir between 10 and 14 h post-challenge. Subsequently, recovery was noted on day 2, whereas controls exhibited a robust circadian profile
Boivin et al. [26]	Estimation of clock gene oscillations in human blood mononuclear cells derived from three human volunteers	Presence of circadian oscillations of *Per 1*and *Per 2* genes
Haimovich et al. [27]	Assessment of clock gene alterations upon LPS administration in peripheral human blood leucocytes, after challenging them with *in vivo* endotoxin or saline, either at 09:00 a.m or 09:00 p.m.	LPS induced a profound suppression of all clock gene expression by 80% to 90%, between 13 and 17 h post-perfusion, whereas IL-6 and TNF-α returned to baseline within 6 h. However, melatonin and cortisol circadian rhythms were not affected by LPS challenge
Pontes et al. [32]	Colostrum samples for measuring tumor necrosis factor α (TNF-α) and melatonin content were collected from 18 normal delivered mothers in the morning, and diurnal and nocturnal melatonin levels in colostrum from healthy puerperae and mothers with mastitis were compared	Suppression of nocturnal melatonin rise in mothers with mastitis was highly correlated with increased tumor necrosis factor α secretion.
		On the other hand, stimulated, but not quiescent, immune-competent cells secreted in the colostrum produced melatonin *in vitro*. In addition, this production ceased after bacteria killing
Cruz-Machado et al. [33]	Effects of LPS on melatonin production in rat pineal cultures	Shutdown of melatonin production through TNF-α induction of NF-kB in pineal microglial cells

##### The immune-pineal axis

A continuous communication between the pineal gland and the immune response has been suggested to exist, defining the ‘immune-pineal axis’ [29]. Thus, pineal melatonin nocturnal secretion enhances Th1/Th2 ratio within low ‘chronobiotic’ levels (nM-pM range) and inhibits at the same time both rolling and adherence of leucocytes to the endothelial layer, decreasing unnecessary inflammatory response [29–31]. Furthermore, extrapineal melatonin produced by local immune-competent cells acts in a paracrine manner as anti-inflammatory mediator in higher concentrations (mM range) [32–34]. Thus, it seems that in the early phase of inflammation, the body does not receive circadian information through the hormonal arm.

Markus et al. [35] have postulated that systemic inflammation activates the nuclear factor kappa B (NF-kB) pathway through LPS/TLR4 signaling at the level of pinealocytes and suppresses central melatonin nocturnal secretion, enhancing migration of immune cells at the site of injury. At the same time, different inflammatory mediators upregulate melatonin production in peripheral macrophages. This extrapineal tissue melatonin has been described as ‘immune buffer’ since it seems to play a dual role [36]. During acute stress, it acts as immunostimulant, improving bacterial phagocytosis, and subsequently, it enhances recovery phase by inducing production of anti-inflammatory cytokines. However, during an exacerbated inflammatory response, melatonin acts mainly as an anti-inflammatory molecule.

Corticosteroids may also affect melatonin pineal production [37, 38]. Thus, by inhibiting the NF-kB pathway in the pineal gland, they can restore its nocturnal rise [37] and enhance its production in a bell-shaped manner [38]. However, they can also decrease the activity of *N*-acetyltransferase (NAT) which is a key enzyme in the biosynthetic pathway of melatonin and hence inhibit its pineal production [39]. Finally, increased cortisol response to stress has been correlated with decreased amplitude of its own circadian rhythm [40].

In summary, different experimental studies confirm the existence of circadian oscillations of the immune response, which can be significantly suppressed by LPS. In addition, mortality seems to depend on time of LPS administration (Table 1).

#### Circadian rhythm profiles and critical illness

##### Circadian output assessment

**Figure 2:**
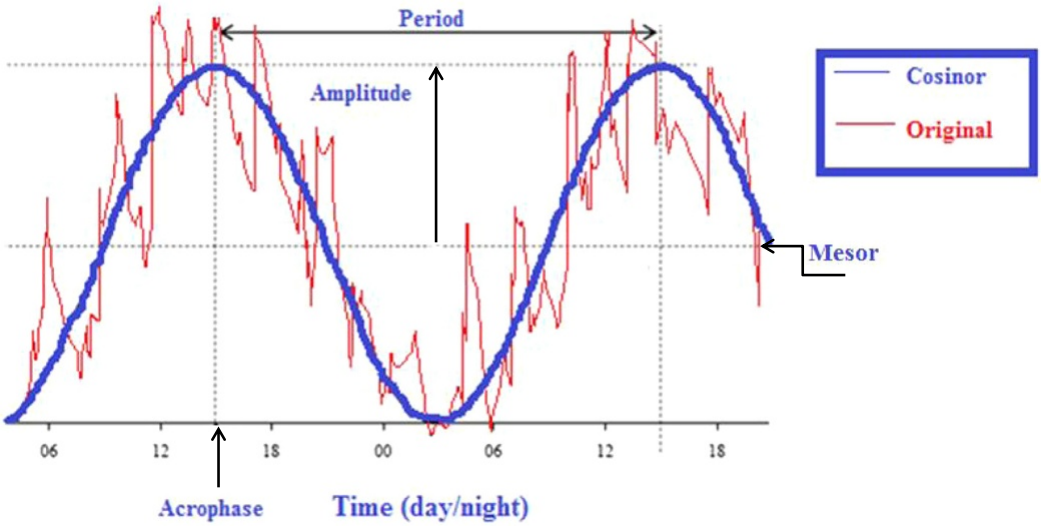
**Chronobiologic analysis of a time series through cosinor analysis.** Schematic illustration of basic metrics derived from cosinor analysis: This method is applicable to the individual biological time series anticipated to be rhythmic with a given period. The procedure fits a cosine function (blue) to the data (red) by least squares. Midline estimating statistic of rhythm (MESOR) is the mean level of oscillation that is the average value of the rhythmic function (e.g., cosine curve) fitted to the data. Amplitude is the difference between the maximum and the MESOR. Acrophase is the time of occurrence of the maximum value.

Periods and modeling variability of different biological time series that reflect circadian output, such as melatonin and cortisol, are assessed via cosinor analysis [41]. Briefly, this technique fits a cosine function of a fixed anticipated period to the data and approximates the following equation to experimental data, using the least squares method for minimization:
1

where, *M* is the midline estimating statistic of rhythm (MESOR), the mean level of oscillation; *A* is the amplitude, the extent of oscillation from the MESOR or half of the total oscillation; π is 3.14159; TAU is the chosen period; *t* is a temporal fraction of the cycle, an instant of the whole revolution; and Φ (phi) is the acrophase, lag from a defined reference time point (e.g., local midnight when the fitted period is 24 h) of the crest time in the cosine curve fitted to the data (Figure 2).

Except for serum melatonin, its urine metabolite 6-sulfatoxymelatonin (6-SMT) [10] and core body temperature (CBT) [42] are accepted biomarkers of circadian rhythm in critically ill patients.

##### Circadian disruption in critically ill patients

Circadian rhythms are disrupted by illness and intensive care unit (ICU) environment, associated with patient care interactions and unregulated light/dark patterns. Different clinical studies have demonstrated that a significant proportion of critically ill patients display long-term sleep disturbance and metabolism, suggesting a contribution of biological rhythm alterations [43, 44].

In this respect, many authors have investigated circadian biomarkers in different groups of patients during their ICU stay, in order to assess a potential circadian dysfunction during critical illness (Table 2) [45–51]. Such misalignment occurs when there is an alteration between cycle frequency and phase in two or more rhythms [5, 12]. Its clinical significance has been established in different settings, since it has been shown to induce a prediabetic condition in healthy humans [52] and symptoms associated with heart failure in animal models of cardiovascular disease [53]. Furthermore, misaligning the cortisol rhythm has been shown to induce profound cardiovascular and renal disease sequel, which was subsequently reversed by light exposure therapy in hamsters [54].

**Table 2:** **Circadian disruption in critically ill patients: clinical studies**

Author	Study design	Major outcome
Tweedie et al. [45]	Retrospective study for characterizing core body temperature (CBT) 24-h profiles of 15 ICU patients	80% of all patient days had a significant circadian rhythm with erratic acrophases and normal amplitudes
Nuttall et al. [46]	Retrospective study assessing clinical significance of circadian rhythms in patients with (≤17) and without (*n* = 120) ICU psychosis, by comparing for 24 h the time of both temperature and urine output nadir	Both groups had altered circadian rhythms, and although all ‘patient days’ had a significant rhythm, 83% of those days had abnormal cosinor-derived parameters
Olofsson et al. [47]	Study of melatonin levels in both blood and urine in 8 critically ill patients under sedation and mechanical ventilation	The circadian rhythm of melatonin release was abolished in all but 1 patient, whereas no correlation was found between melatonin levels and level of sedation
Frisk et al. [48]	Study of 6-SMT and urine cortisol in 16 patients, treated in the ICU of two regional hospitals	Hyposecretion of 6-SMT during mechanical ventilation, increase upon adrenergic stimulation, overall high cortisol excretion and, finally, a disturbed diurnal rhythm of both these hormones in 75% of all patients
Paul and Lemmer [49]	Measurement of CBT every hour and plasma cortisol and melatonin levels every 2 h for 24 h, in 13 sedated ICU patients following surgery or respiratory failure and 11 patients with brain injury	The 24-h circadian profiles of all measured variables were significantly disturbed, with no physiological day-night rhythm in both groups of patients in relation with healthy controls, whereas circadian rhythm alterations were more pronounced in patients with brain injuries
Pina et al. [50]	Prospective analysis of hourly CBT and 4-h interval urine cortisol, melatonin, and 6-SMT profiles in 8 burn patients and 14 controls for 24 h in three sessions, occurring between ICU days 1 to 3, day 10, and days 20 to 30	Circadian rhythms of all measured variables were abolished in all patients in relation with controls. Burn ICU patients displayed significantly higher MESORS of CBT, urine melatonin, 6-SMT, and cortisol compared with the control group, during the three sessions of measurements. 24-h circadian profiles were restored within a 30-day period
Gazendam et al. [51]	Investigation of circadian rhythm disruption in a general ICU population, assessed using CBT profiles over a 48-h period in 21 patients	Acrophase shift in all cases. Acute Physiology and Chronic Health Evaluation (APACHE) III score was predictive of circadian misplacement
Mudlinger et al. [55]	Circadian alterations in 17 septic patients versus 7 non-septic subjects and 21 controls, in the ICU	Urinary 6-SMT exhibited circadian rhythmicity in only 1 of 17 septic patients versus 6 of 7 in non-septic patients and 18 of 23 in normal controls. MESORS appeared slightly increased, phase amplitudes were markedly lower, and acrophase occurred later in septic patients. On the contrary, in both non-septic patients and controls, 6-SMT exhibited a circadian rhythm
Perras et al. [56]	Measurement of single nocturnal melatonin concentration (NMC) in 302 patients during their first night in ICU	Analysis of the whole study population did not reveal any correlation between single melatonin measurement and APACHE II score, but in 14 patients with severe sepsis, an inverse correlation was found
Bagci et al. [57]	Nocturnal plasma melatonin and 6-SMT urine concentrations were measured in 23 septic and 13 non-septic pediatric ICU patients	The NMC during septic shock was increased in relation with no shock states. There was no difference for nocturnal and total 6-SMT excretion between septic patients with and without septic shock and non-septic patients. Nocturnal and total 6-SMT excretion was significantly lower in septic patients with than in septic patient without liver dysfunction. Sedation and mechanical ventilation did not affect melatonin excretion
Gehlbach et al. [58]	Assessment of sleep/wake regulation and circadian rhythmicity for 24 h, through 1-h interval urine measurements of 6-SMT, in 22 mechanically ventilated patients with different diagnoses of ICU admission	The 24-h temporal profile of 6-SMT exhibited a phase delay. There was no difference between patients with and without sepsis and no correlation between APACHE II score and 6-SMT amplitude
Li et al. [59]	11 septic and 11 non-septic patients in ICU. Peripheral blood was drawn at 4-h intervals during the first day of admission	The melatonin secretion acrophase occurred earlier in septic patients compared with non-septic patients. Melatonin MESORS tended to be higher in the septic group. Both Cry-1 and Per-2 expression were decreased, while TNF-α and IL-6 expression were increased in septic patients, reaching a peak at 6:00 p.m, which was consistent with the altered rhythm of melatonin secretion. Suppression of peripheral circadian genes was independent of the melatonin rhythm
	Plasma levels of melatonin, TNF-α, IL-6, and messenger RNA levels of circadian genes Cry-1 and Per-2 were analyzed	

Only a few investigators have evaluated circadian alterations during sepsis (Table 2) [55–59]. Mudlinger et al. [55] assessed in ICU circadian disruption in 17 septic patients versus 7 non-septic and 21 controls. Urinary 6-SMT, measured at 4-h intervals over a 24-h period, exhibited significant loss of circadian rhythmicity with no daytime decline in septic versus non-septic patients and controls, respectively.

Recently, Li et al. [59] studied for 24 h 11 septic and 11 non-septic ICU patients and measured during the first day of admission plasma levels of melatonin, TNF-α, and IL-6 and messenger RNA levels of circadian genes Cry-1 and Per-2. The authors found altered circadian rhythm of melatonin secretion, decreased expression of both Cry-1 and Per-2, and high levels of TNF-α and IL-6 in septic patients. They also showed that the suppression of peripheral circadian genes was independent of the melatonin rhythm.

In conclusion, Li et al. [59] confirmed that during acute phase of sepsis in humans, there is an uncoupling of the central master clock and peripheral tissue-specific clock genes, associated with pro-inflammatory cytokine production. Moreover, acrophase shift exhibited an advance rather than a delay in septic patients, contrary to what was found in the study of Mudlinger that included patients with at least 1 week stay in the ICU [55]. We suppose that at the early stages of sepsis, the inverse relation between melatonin and pro-inflammatory cytokines that was clearly shown in different animal models is more evident [35]. However, during the late stages, medications, such as catecholamines and varying levels of sedation [48, 60], could also alter circadian rhythms, since both morphine [60] and benzodiazepines [48] have been shown to induce NAT activity and enhance in a dose-dependent manner daytime production of melatonin [10]. In addition, mechanical ventilation [48] and ICU milieu [61–63] may further disrupt circadian variations, limiting accurate assessment of immune-circadian connectivity.

##### ICU environment and circadian output disruption

ICU milieu can be considered as a particular stress trigger for the internal circadian clock. Exposure to persistent environmental light has been recognized as a serious concern in the ICU [61–63]. However, different authors have found that light failed to influence circadian rhythms in healthy subjects [64] and in septic critically ill patients under controlled ventilation [65, 66], suggesting that sepsis *per se* could decrease sensitivity to light exposure.

Drugs are potential confounders of immune-circadian connectivity in critically ill patients. In this respect, both opioids [50] and benzodiazepines [67] may alter melatonin production. Additionally, increased sympathetic tone and use of vasopressors during septic shock could theoretically enhance melatonin excretion. However, sympathetic reuptake of norepinephrine [68] and poor responsiveness of human pineal gland to circulating catecholamines [69] protect against the inappropriate increase in pineal melatonin production.

Another significant stressor of circadian rhythms in sedated patients is the sleep/wake cycle disruption. Different studies have confirmed that the majority of these patients experience either sleep deprivation and/or sleep fragmentation [43, 44, 58, 70]. It has been suggested that the dispersion of episodic ‘sleep-like states’ could be responsible for the reduced amplitude and acrophase delay of urine 6-SMT that was also noticed in healthy subjects [58, 71]. Nevertheless, it seems that sleep *per se* remains a weak timekeeper in humans without a concomitant change in the light/dark cycle [72]. Finally, delirium has been implicated as a pathologic state modifying melatonin excretion in elderly conscious medical patients [10, 73]. At the same time, melatonin circadian deregulation has been associated with neurotransmitter alterations and subsequent delirium in septic patients [74, 75]. However, it remains unclear if it is the quantity or the rhythm profile of melatonin that is related to delirium occurrence [10].

### Potential therapeutic implications

Duboule [76] and Halberg [77] introduced the term ‘chronomics’, time and rhythm, for describing circadian regulation of animal development and chronotherapy in different disease states. Evidence from observational studies is growing that circadian disruption contributes to the development of cancer [76, 78]. So, it has been suggested that melatonin could be beneficial in cancer treatment when administered at chronobiologically determined optimum times of the day [79].

Administration of melatonin has been found in both animals [79, 80] and one human study in neonates [81] to reduce hyperinflammatory response during sepsis. In addition, it has been shown that melatonin exhibits an *in vitro* antimicrobial activity against multi-drug resistant Gram-negative and Gram-positive bacteria due to free iron binding [82] and furthermore can protect kidney grafts from ischemia-reperfusion injury [83]. Moreover, prolonged nighttime melatonin administration lowers blood pressure in hypertensive subjects [84], since SCN neurotransmitter content and transmission are suppressed during hypertension [85]. Finally, in two randomized placebo-controlled trials, melatonin [86] and a synthetic analog [87] were found to decrease incidence of delirium in elderly medical patients, but did not affect its duration or severity. However, intention-to-treat analysis was not possible in the first trial because of lost to follow-up patients.

Nevertheless, many aspects remain unresolved. Thus, prior knowledge of the circadian profile of the patient is needed in order to design a personalized melatonin dose and duration of treatment, as well as chronobiologically determined optimum time of administration, since a circadian rhythmicity has been found for both pharmacokinetics and pharmacodynamics of different drugs, such as antibiotics [88]. Furthermore, melatonin excretion can be altered by liver and renal injury or by circadian modulation of hepatic function and glomerular filtration rate [10, 89]. In this respect, different timekeepers, such as light or medications, have been used in cancer or psychiatric disorders, on the right time and order and at a specific phase of the circadian cycle [78, 90]. Similarly, different ‘rhythm therapies’ could be scheduled for ICU patients, following the *kairos* principle (right time of the day) instead of *chronos* (time in general) [76, 78]. Moreover, introduction of additional timekeepers and excitation of the biological system with ultradian short-period rhythms, such as light or art therapy, have been found to enhance long-period fluctuations of melatonin by excitation, coupling, and resonance [91]. As a result, a restored circadian rhythmicity has been noticed in patients with sleep disorders and subjects with jet lag [91]. Such effects may also enlarge the circadian cycle of heart rate variability (HRV), which is connected with sleep quality and ANS dysfunction [78, 92].

It has been postulated that entrained and synchronized circadian rhythms better prepare the physiology of an individual to anticipate normal cycles of energy demand in order to optimize adaptive regulation [93]. This ‘self-adaptation’ behavior is transformed into a ‘self-defense’ response during stress [31], explaining results from different studies. Thus, pro-inflammatory cytokines shut down melatonin's nocturnal surge in the acute phase, whereas exacerbated or chronic inflammation upregulates pineal production through anti-inflammatory mediators, such as corticosteroids [36, 38]. However, there is a lot of heterogeneity in different studies due to interspecies differences or time and severity of inflammatory insult, prompting a standardization of experimental protocols for translating results in the ICU setting.

Since severity of disease varies across the day and night [20, 94] and the temperature curve might exhibit an inverted pattern (febris inversa) in different infections, such as tuberculosis where fever is higher in the morning than in the evening, we suggest that future studies should assess differences in terms of circadian profiles, between patients suffering from an inflammatory episode that occurs at different time points of a 24-h period. Moreover, and since light unresponsiveness of SCN has been found in septic patients [65, 66], we suppose that in this particular group, possible circadian misalignment might reflect mainly individualized immune-circadian connections. In that case, it would be interesting to study if different circadian biomarkers correlate significantly with the Sequential Organ Failure Assessment (SOFA) score of severity of illness and predict mortality better than SOFA. In addition, ICU environmental profiles could be correlated with trajectories of circadian biomarkers, and different environmental approaches to patient care, such as ‘virtual darkness’ by shortening the day length, could be designed and tested to promote more rapid attainment of circadian rhythms [95]. Finally, restoring circadian light/dark cycle might improve immune function through enhanced melatonin production, in the context of reduced energy availability associated with critical illness, as is currently observed in lower mammals during the winter [95].

Except for clinical researchers, basic scientists could also benefit from chronobiological analytic tools in order to design experimental studies and assess treatment effects in different septic models. It has been recognized that some of the reasons for negative results in different clinical trials in septic patients [96], despite encouraging results from preclinical studies, are the use of animal models that do not adequately mimic human sepsis [97]. Furthermore, misinterpretation of preclinical data or adoption of different experimental protocols has been considered as a contributing factor for this discrepancy [97]. In this respect, the use of ‘higher fidelity animal models’ has been suggested in order to increase the clinical relevance of experimental research [98]. Nevertheless, we would like to highlight the importance of assessing immune-circadian connectivity as a further step for translating basic science results into successful randomized controlled trials. Thus, different models should evaluate clock gene expression in immune-competent cells upon LPS challenge at standardized time points and in different environmental settings (i.e., light manipulation) [99], whereas clock gene knockout animals could also be used for assessing circadian-immune disconnection. Finally, new statistical methods, such as EUCLIS (EUCLOCK Information System, an EU FP6 project) [100] could be tested for analyzing the genome, the proteome, and the metabolome.

## Conclusions

As was suggested by Haldberg et al. [77], ‘in biologic time series that are dense and sufficient long the characteristics of rhythms and trends can be quantified as elements of structures called chronoms’. ‘Microscopy-in-time’ chronobiology studies cycles in biological time series with mechanisms embedded in living matter, whereas ‘telescopy-in-time’ chronomics assesses their alignment with environmental cues [101]. Thus, chronobiologic surveillance could be implemented in the ICU, serving a better understanding of biologic complexity in critical illness and, subsequently, an individualized optimization of treatment. In this respect, vascular variability anomalies (VVAs) estimated with chronomics, such as heart rate and blood pressure variability, have been recognized as significant risk factors in patients with cardiovascular diseases [102]. Similarly, reduced HRV has been repeatedly demonstrated in patients with sepsis and organ dysfunction [28]; however, chronobiologic analysis has not been performed so far.

In the context of negative results from different clinical studies in septic patients [96], we suggest that individual rhythm analysis might add significant value to the caring of critically ill. Thus, continuous monitoring of different biosignals, such as electrocardiogram (ECG), could detect diurnal variations in HRV and patterns of change specific for each patient and each pathophysiological state, creating an individual profile of ‘physiomarkers’ that could be used as both a diagnostic and therapeutic monitoring tool in everyday clinical practice. In addition, circadian aspects of pharmacokinetics and both liver and renal function could be considered in daily treatment, in order to increase efficiency and/or reduce adverse effects of medical therapy on a personalized basis. Finally, future clinical trials should assess circadian aspects of immunity and therapeutics for evaluating treatment effects. In this respect, adoption of different modeling techniques and design of *in silico* studies could be applied towards understanding inflammation and translate computational systems biology approaches in sepsis research to clinical relevance [103].

## Abbreviations

ANS: autonomic nervous system
APACHE: Acute Physiology and Chronic Health Evaluation
CBT: core body temperature
CVO: circumventricular organs
EUCLOCK: Entrainment of the circadian clock
ECG: electrocardiograph
EU FP6: European Union frame project 6
HRV: heart rate variability
ICU: intensive care unit
IL-6: interleukin-6
INF-γ: interferon-γ
LPS: lipopolysaccharide
MESOR: midline estimating statistic of rhythm
NAT: *N*-acetyltransferase
NMC: nocturnal melatonin concentration
PVN: paraventricular nucleus
SCN: suprachiasmatic nucleus
6-SMT: 6-sulfatoxymelatonin
SOFA: Sequential Organ Failure Assessment
TLR: toll-like receptor
TNF-α: tumor necrosis factor α
VVA: vascular variability anomalies.
